# Ultra-Processed Foods Consumption and Metabolic Syndrome in European Children, Adolescents, and Adults: Results from the I.Family Study

**DOI:** 10.3390/nu17132252

**Published:** 2025-07-07

**Authors:** Annarita Formisano, Marika Dello Russo, Lauren Lissner, Paola Russo, Wolfgang Ahrens, Stefaan De Henauw, Antje Hebestreit, Timm Intemann, Monica Hunsberger, Dénes Molnár, Luis Alberto Moreno, Valeria Pala, Stalo Papoutsou, Lucia Reisch, Toomas Veidebaum, Garrath Williams, Maike Wolters, Alfonso Siani, Fabio Lauria

**Affiliations:** 1 Institute of Food Sciences, National Research Council, 83100 Avellino, Italy; annarita.formisano@isa.cnr.it (A.F.); marika.dellorusso@isa.cnr.it (M.D.R.); paola.russo@isa.cnr.it (P.R.); asiani@isa.cnr.it (A.S.); 2 Department of Public Health and Community Medicine, University of Gothenburg, SE 413 90 Gothenburg, Sweden; lauren.lissner@gu.se (L.L.); monica.hunsberger@gu.se (M.H.); 3 Leibniz Institute for Prevention Research and Epidemiology-BIPS, 28359 Bremen, Germany; ahrens@leibniz-bips.de (W.A.); hebestr@leibniz-bips.de (A.H.); intemann@leibniz-bips.de (T.I.); wolters@leibniz-bips.de (M.W.); 4 Department of Public Health and Primary Care, Faculty of Medicine and Health Sciences, Ghent University, 9820 Ghent, Belgium; stefaan.dehenauw@ugent.be; 5 Department of Pediatrics, Medical School, University of Pécs, 7624 Pécs, Hungary; molnar.denes@pte.hu; 6 Growth, Exercise, Nutrition and Development (GENUD) Research Group, University of Zaragoza, C/Domingo Miral s/n, 50009 Zaragoza, Spain; lmoreno@unizar.es; 7 Instituto Agroalimentario de Aragón (IA2), C/Miguel Servet 177, 50013 Zaragoza, Spain; 8 Instituto de Investigación Sanitaria de Aragón (IIS Aragón), Avda. San Juan Bosco 13, 50009 Zaragoza, Spain; 9 Centro de Investigación Biomédica en Red-Fisiopatología de la Obesidad y Nutrición (CIBEROBN), (CB15/00043), Institute of Health Carlos III (ISCIII), Av. Monforte de Lemos, 3-5, Pabellón 11, Planta 0, 28029 Madrid, Spain; 10 Department of Preventive and Predictive Medicine, Fondazione IRCCS, Istituto Nazionale dei Tumori, 20133 Milan, Italy; valeria.pala@istitutotumori.mi.it; 11 Research and Educational Institute of Child Health, 2031 Strovolos, Cyprus; stalo.papoutsou@gmail.com; 12 El-Erian Institute for Behavioural Economics and Policy, Cambridge Judge Business School, University of Cambridge, Cambridge CB2 1AG, UK; lr540@cam.ac.uk; 13 National Institute for Health Development, Tervise Arengu Instituut, 10617 Tallinn, Estonia; toomas.veidebaum@tai.ee; 14 Department of Politics, Philosophy and Religion, Lancaster University, Lancaster LA1 4YW, UK; g.d.williams@lancaster.ac.uk

**Keywords:** ultra-processed foods, metabolic syndrome, children, adolescents, adults

## Abstract

**Background/Objectives**: Ultra-processed foods (UPFs) constitute a large proportion of the daily energy intake of Europeans, particularly among children and adolescents. High UPFs consumption is associated with poor dietary quality and adverse health outcomes. This study aimed to examine whether high UPFs consumption is associated with metabolic health in children, adolescents, and adults, using data from the I.Family study. **Methods**: This cross-sectional analysis (2013/2014) included 2285 participants: 147 children (6–9 years), 645 adolescents (10–19 years), and 1493 adults (≥20 years). For the children and adolescents, a metabolic syndrome (MetS) *z*-score was calculated, consisting of age- and sex-standardized *z*-scores of WC, HOMA index, HDL-C, TRG, systolic blood pressure (SBP), and diastolic blood pressure (DBP). For the adults, MetS was defined according to the criteria of the International Diabetes Federation Task Force and other societies. The participants completed at least one 24 h recall, from which their UPFs consumption was estimated using the NOVA classification. The consumption levels were divided into age- and sex-specific quintiles based on the relative energy contribution of these foods. Multivariable regression analyses were conducted to evaluate the associations between UPFs consumption and MetS or its components. **Results**: No statistically significant associations were found between UPFs consumption and MetS or its components in any age group. The effect sizes were negligible across the quintiles (η^2^ = 0.0065 in children, 0.015 in adolescents, and 0.0009 in adults). While the mean MetS score showed little variation, the prevalence of MetS scores above the 90th percentile increased in the highest UPFs quintile among the children. The diet quality decreased with increasing UPFs consumption. **Conclusions**: UPFs consumption was not associated with MetS or its components across the age groups. However, a decline in diet quality was observed with increasing UPFs intake, highlighting the importance of public health strategies to reduce UPFs consumption and improve dietary patterns, particularly among younger populations.

## 1. Introduction

In recent decades, global dietary habits have undergone a significant transformation, influenced by various factors, including socio-cultural changes, industrialization, technological advancements, and the globalization of food production [1]. In many countries, a clear nutritional transition has emerged, characterized by the replacement of traditional foods and freshly prepared meals with an increased consumption of highly processed foods, known as ultra-processed foods (UPFs) [2]. UPFs are products that have been substantially modified from their original form through multiple physical, chemical, or biological processing steps, often including the addition of ingredients, such as preservatives, flavours, nutrients, and other substances approved for use in food products [3]. According to the NOVA food classification system, UPFs are characterized by the inclusion of ingredients and additives not commonly used in home cooking, often intended to enhance palatability, shelf life, or convenience. Their composition and examples of them vary across regions, but share the common characteristic of extensive processing [3]. Designed for immediate consumption and requiring minimal preparation at the time of consumption, these foods offer an extended shelf life, quickness and convenience of preparation, and heightened palatability, making them competitive with whole and freshly prepared foods. However, their nutritional profile is often characterized by high levels of saturated fats, sugar, and sodium, being energy-dense but low in nutrient density, protein, dietary fibre, and micronutrients [4]. Recent findings from the I.Family study have shown that UPFs represent a significant proportion of the daily energy intake of Europeans, particularly children and adolescents [5]. Moreover, it has been shown that high UPFs consumption leads to nutritionally unbalanced and poor-quality diets [5]. Specifically, it is estimated that these foods contribute approximately 50% of the total dietary energy intake, reflecting similar consumption levels to those of other high-income countries, including the USA, Canada, and the UK [5,6,7,8].

Numerous studies have highlighted a strong association between the consumption of UPFs and negative health outcomes. Several cross-sectional and cohort studies have shown that the intake of UPFs plays a significant role in increasing the incidence of non-communicable diseases (NCDs), including obesity and type 2 diabetes, as well as dyslipidaemia and hypertension [8,9,10,11]. Additionally, higher UPFs consumption has been associated with increased mortality from all causes [12]. However, the evidence regarding the relationship between UPFs and metabolic syndrome (MetS) remains limited and, at times, inconsistent, particularly in younger populations, highlighting the need for further investigation. MetS, defined in adults as a cluster of different risk factors—including central obesity, hyperglycaemia, dyslipidaemia, and elevated blood pressure—has increasingly become a significant public health concern globally, paralleling the epidemic of overweight and obesity [13,14]. Studies of adult populations have indicated that a higher consumption of UPFs is associated with an increased risk of developing MetS [15,16]. For instance, a cohort study conducted by Canhada et al. of adults from the ELSA-Brasil study found that a greater intake of UPFs and beverages was independently associated with an increased risk of MetS over an approximately 8-year follow-up period. Increased UPFs consumption was linked to a 19% higher risk of MetS [16]. However, not all the research supports this association. A recent prospective cohort study conducted among Brazilian adults did not identify a significant relationship between UPFs consumption and the risk of MetS [17]. Similarly, a study by Barbosa et al. on women from Quilombola communities in Alagoas reported no significant association between UPFs intake and MetS [18]. In adolescents, evidence of a potential link between UPFs consumption and MetS has also been observed. For example, a cross-sectional study of adolescents aged 12 to 19 years showed a positive association between UPFs consumption and MetS [19]. This relationship was reflected in metabolic alterations, including increased waist circumference, elevated triglyceride levels, and reduced HDL cholesterol [19,20].

Given the existing gaps and inconsistencies in the scientific literature, particularly regarding younger populations, the present study aims to provide clearer insight. The availability of a large cohort of children, adolescents, and adults from eight European countries within the I.Family study provides a unique opportunity to explore these still poorly defined aspects in greater depth. This analysis aims to systematically assess whether a high consumption of UPFs is associated with the prevalence of MetS and its components in a heterogeneous European population, varying in age and socio-cultural characteristics.

## 2. Materials and Methods

### 2.1. Study Population

The data used in this cross-sectional study were derived from the European multicentre I.Family study (http://www.ifamilystudy.eu/, accessed on 20 May 2025). The I.Family study, conducted between 2013 and 2014, employed standardized measures and protocols across eight European countries: Belgium, Cyprus, Estonia, Germany, Hungary, Italy, Spain, and Sweden. The examination involved 7228 children, their siblings (*n* = 2364), and parents (*n* = 7788) [21]. Participants with missing information about any metabolic risk factors, an implausible energy intake (<500 or >3500 kcal/day), or those who did not meet the fasting requirement of at least 8 h were excluded from the analysis. The included and excluded participants were compared in terms of their age, sex, BMI (or BMI *z*-score), and UPFs consumption to assess the potential selection bias.

Before enrolment in the study, written informed consent was obtained from the parents of all the participants. For children under 12 years old, verbal consent was also obtained from the children. Adolescents aged 12 years or older signed a simplified consent form. Ethical approval was obtained from the institutional review boards of all the participating centres. All the procedures were carried out in compliance with the ethical guidelines outlined in the Declaration of Helsinki and its subsequent revisions.

### 2.2. Dietary Information

Information on the participants’ dietary intake was collected using the validated web-based 24 h dietary recall (24-HDR) tool, *Self-Administered Children*, *Adolescents and Adult Nutrition Assessment* SACANA [22,23]. A detailed description of the SACANA software was provided in a previous study [22]. The participants were asked to complete multiple 24-HDRs, including at least two for weekdays and one for a weekend day, within the two weeks following the initial 24-HDR completed with assistance at the examination centre. All the subjects included in this analysis completed at least one 24-HDR. The children aged 11 years and older completed the 24-HDR independently, while parents were asked to assist younger children in filling it out [24].

Using the 24-HDR, the participants reported their dietary intake for the previous 24 h, specifying the type and quantity (in grams) of all foods and beverages consumed, from the first intake after waking up until the last intake before going to sleep. Standardized food images were provided to enhance accuracy in estimating portion sizes. The total energy and nutrient intake for each food or recipe were calculated using the German Food Composition Database, Bundeslebensmittelschlüssel (BLS 3.02 version), chosen for its laboratory-analysed foods and comprehensive nutritional information, ensuring greater uniformity and comparability at the international level [25]. With regard to this study, it is important to add that the BLS is used by the food industry for calculating the mandatory nutrition labelling in accordance with the requirements of the Food Information Regulation (EU) No. 1169/2011 [26]. According to the NOVA classification system, each food and beverage reported on the 24-HDR was categorized into one of the following four groups based on the extent and purpose of its industrial processing: 1—unprocessed or minimally processed foods (e.g., fresh fruits and vegetables, eggs, milk, and unprocessed meat); 2—processed culinary ingredients (e.g., vegetable oils, butter, and honey); 3—processed foods (e.g., canned or bottled vegetables and legumes, canned fish, and freshly made bread); and 4—UPFs (e.g., soft drinks, processed meat, instant packaged soups, biscuits, sweet or savoury packaged snacks, and sugared milk and fruit drinks) [27]. The classification of each item was independently reviewed by three co-authors, with discrepancies resolved by consensus through discussion. We focused our analysis on the fourth NOVA group classification, which represents UPFs. Details of the classification process have been described in our previous publication [5]. Individuals’ usual energy intake from the principal macronutrients in the UPFs NOVA group was estimated based on the validated National Cancer Institute method for children, adolescents, and adults, separately [28]. Additionally, the relative energy contribution of the UPFs to the total energy intake was calculated. The percentage contributions of protein, fat, saturated fatty acids, carbohydrates, and sugars to the total energy intake (%TEI) were determined, while the fibre intake was expressed as grams per 1000 kcal per day. In the end, based on the relative energy contribution, the UPFs group was divided into age- and sex-specific quintiles.

The Healthy Diet Adherence Score (HDAS) was used to assess the extent to which individuals adhered to healthy dietary recommendations, which suggest a higher intake of whole grains, fruits, vegetables, and fish, while reducing the consumption of fats and refined sugars [29]. The score ranges from 0 to 50, with higher values reflecting greater compliance with these dietary recommendations [29,30]. This score was developed using the Food Frequency Questionnaire (FFQ) section of the Children’s Eating Habits Questionnaire (CEHQ-FFQ) [30].

### 2.3. Physical Measurements and Laboratory Analyses

The anthropometric measurements were performed by trained personnel following standard operating procedures in line with international standards, including intra- and inter-observer reliability, as previously documented [31]. The measurements were taken under fasting conditions, with the participants wearing light clothing and standing barefoot. Height was measured to the nearest 0.1 cm using a calibrated stadiometer (SECA 225, Birmingham, UK), and body weight was measured to the nearest 0.1 kg using an electronic balance (Tanita BC 420 SMA, Tanita Europe GmbH, Sindelfingen, Germany). The body mass index (BMI) was calculated as weight in kilograms divided by the square of height in meters. Age- and sex-specific BMI *z*-scores were derived for the children and adolescents, and categorized into groups (normal weight, overweight, or obese) according to the International Obesity Task Force (IOTF) criteria [32]. For the adults, the BMI categories were based on standard international classifications [33]. The waist circumference (WC) was measured at the end of normal expiration, at the midpoint between the superior iliac crest and the costal margin, using an inelastic tape (SECA 200, Hamburg, Germany) with a precision of 0.1 cm, while the subject was standing.

The systolic (SBP) and diastolic blood pressure (DBP) were measured using an automatic sphygmomanometer (Welch Allyn 4200B-E2, Welch Allyn Inc., Skaneateles Falls, NY, USA) with an appropriately sized cuff. The participants were asked to sit quietly for at least five minutes prior to measurement. Two readings were taken with a two-minute interval, and an additional reading was taken if the difference between the first two measurements exceeded 5%.

Fasting venous blood samples were collected voluntarily to measure blood glucose, total cholesterol, high-density lipoprotein cholesterol (HDL-C), and triglycerides (TRG), following standardized procedures previously reported [21]. The homeostatic model assessment (HOMA) index was calculated according to Matthews et al. [34]. To normalize the values for the statistical analysis, the sex- and age-specific *z*-scores were computed for the WC, SBP, DBP, HOMA index, TRG, and HDL-C in the children and adolescents [35,36,37,38].

### 2.4. Metabolic Syndrome and Its Components

MetS in the children and adolescents was assessed using the approach proposed by Ahrens et al. [39]. This method considers a MetS score, representing the cluster of components typically used to define MetS in adults. Specifically, the score was calculated by summing the age- and sex-standardized *z*-scores of the WC, HOMA index, HDL-C, TRG, systolic blood pressure (SBP), and diastolic blood pressure (DBP), following the formula outlined by the authors of [39]. In our analysis, unfavourable levels of MetS and its components were identified using a cut-off of ≥90th percentile [39].

In the adults, MetS was defined according to the harmonized definition of Alberti et al. [13], whereby participants were classified as having MetS if they had three or more of the following five cardiometabolic risk factors: (1) elevated TRG levels (≥150 mg/dL); (2) low HDL-C levels (<40 mg/dL for men, <50 mg/dL for women); (3) elevated blood pressure (systolic ≥ 130 mmHg and/or diastolic ≥ 85 mmHg); (4) elevated fasting glucose levels (≥110 mg/dL); and (5) elevated WC (≥102 cm for men, ≥88 cm for women).

### 2.5. Socio-Economic Variables

The parents were asked to provide information about their educational attainment and household income through specific questionnaires. Educational attainment was self-reported by parents based on the International Standard Classification of Education (ISCED), categorized as follows: low (ISCED levels 1–2), medium (ISCED levels 3–4), and high (ISCED levels 5–6) [40]. Household income was reported as the monthly net income (after taxes and deductions) and classified into five categories: low, low–medium, medium, medium–high, and high.

### 2.6. Statistical Analysis

All analyses were performed by using the quintiles of UPFs consumption, expressed as the percentage contribution to total energy intake (%TEI), and stratified by three age groups: 6–9 years, 10–19 years, and ≥20 years. The baseline descriptive characteristics were reported as the means and standard deviations (SDs) for the continuous variables and as percentages for the categorical variables.

The relationship between each quintile of UPFs consumption and metabolic syndrome (MetS) and its components was assessed using an analysis of covariance (ANCOVA), with the estimated marginal means reported and Bonferroni post hoc tests applied for multiple comparisons. The partial eta-squared (η^2^) was used as a measure of the effect size. A preliminary unadjusted (crude) analysis was performed, followed by an adjusted analysis controlling for sex, age, country of origin, BMI, family income, family ISCED, and total daily energy intake.

A binary logistic regression analysis was used to estimate the odds ratios for MetS and its components across the quintiles of UPFs consumption for all age groups. The models were adjusted for the same covariate as in the ANOVA. IBM SPSS Statistics (Version 23.0. IBM Corp., Armonk, NY, USA) was used for the statistical analyses, and statistical significance level was set to alpha = 0.05 (i.e., *p* value < 0.05).

## 3. Results

**Figure 1:**
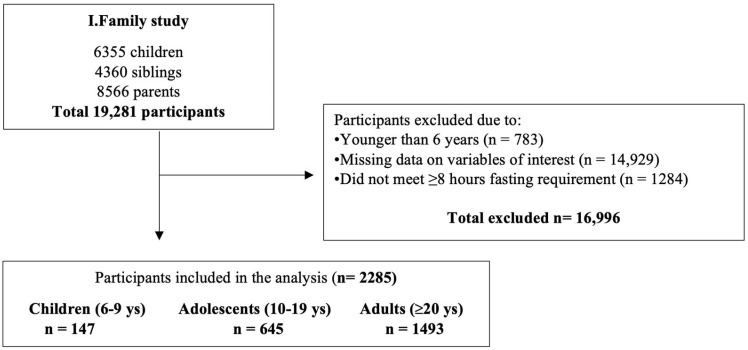
Flow chart for participants included in the final analysis.

The present analysis included 2285 participants (147 children aged 6–9 years, 645 adolescents aged 10–19 years, and 1493 adults aged ≥ 20 years). The flow chart for the selection process is shown in Figure 1.

Table 1 presents the characteristics of the study population, stratified by age group (children, adolescents, and adults). The participants excluded from the analysis differed significantly from those included in terms of their age, BMI, and UPFs consumption. More specifically, significant differences were observed for the age and BMI *z*-scores of the children, for all the variables considered for the adolescents, and for the age and UPFs consumption of the adults. Nonetheless, the differences in the UPFs consumption between the included and excluded individuals were modest: among the adolescents, it was 48.5 ± 9.6 vs. 47.5 ± 10.1 (*p* = 0.013), and among the adults, it was 39.8 ± 9.5 vs. 40.5 ± 9.0 (*p* = 0.032).

The characteristics of the study population across the quintiles of UPFs consumption, expressed as a percentage of the total energy intake (%TEI), are presented in Table S1A–C as Supplementary Table S1. Among the children, the BMI *z*-scores were similar between the highest and lowest quintiles of UPFs consumption, while the waist circumference (WC) *z*-scores were higher for those in the highest quintile. Additionally, a higher proportion of children in the highest quintile were overweight, and they had lower HDL *z*-scores compared to those in the first quintile (Table S1A). Among the adolescents, the participants in the highest quintile of UPFs consumption had lower BMI and WC *z*-scores than those in the lowest quintile, and were predominantly of normal weight (Table S1B). In the adults, the individuals in the fifth quintile of UPFs consumption showed higher triglyceride levels and higher educational attainment compared to those in the first quintile (Table S1C). In both the children and adolescents, the mean MetS score showed little variation across the UPFs quintiles, with no clear pattern observed (Table S1A,B). Regarding the MetS score prevalence above the 90th percentile, it increased among the children in the highest quintile (Table S1C). The diet quality, assessed by the HDAS score, declined progressively with increasing UPFs consumption in the children, adolescents, and adults, although the decline was less pronounced in the adults.

Table 2A–C present the results of the analysis of variance (both crude and adjusted) for the children, adolescents, and adults, respectively. Both the crude and adjusted models consistently found no statistically significant associations between the UPFs consumption and MetS or its individual components across all age groups. The effect sizes across the UPFs consumption quintiles were negligible in the children (η^2^ = 0.0065), adolescents (η^2^ = 0.015), and adults (η^2^ = 0.0009), indicating minimal variation in MetS by UPFs intake.

Finally, the results of the binary logistic regression analysis are presented in Table 3A–C. These findings indicate no association between the dietary proportion of UPFs and MetS or with its individual components, among the children, adolescents, or adults.

Across all the age groups, higher UPFs consumption was statistically significantly associated with higher intakes of total energy, carbohydrates, fats, and saturated fatty acids (SFAs), and with a lower %TEI from protein and fibre. UPFs contributed 48.6% of the total energy intake in children, 47.5% in adolescents, and 40.5% in adults (Table 1).

## 4. Discussion

In this study, we investigated the association between UPFs consumption and the prevalence of MetS in a large multicentre European cohort comprising children, adolescents, and adults from eight countries. The absence of meaningful differences in the incidence of MetS across the UPFs quintiles, supported by the very small effect sizes for all age groups, strengthens the conclusion that UPFs consumption was not associated with metabolic syndrome in this population. Although the BMI is not a component of MetS, it is closely related to metabolic risk. In our analysis, the BMI did not show a consistent trend across the quintiles of UPFs intake. However, we also adjusted for the BMI to ensure that the associations observed for UPFs were independent of the BMI itself.

Our results align with those of previous research that have not found a clear association between UPFs consumption and MetS in similar contexts, reflecting the complexity of the interactions between diet and metabolic health and, perhaps also, the limitations of dietary data [4,18].

Our findings therefore contrast with those of studies that have reported an increased risk of MetS with higher UPFs consumption [15,16,19]. The differences in the findings across studies on UPFs consumption and health outcomes may reflect variability in the UPFs classification methods, population characteristics, study designs, confounder adjustment, and outcome definitions. In particular, differences in dietary assessment tools, cultural dietary patterns, follow-up duration, and the definition and measurement of metabolic syndrome could contribute to inconsistent results. Based on the descriptive analysis, which shows a decrease in the HDAS with an increasing UPFs quintile, these results are consistent with our previous findings, which demonstrated an association between high UPFs consumption and lower diet quality. While this study does not formally assess the relationship between UPFs intake and the HDAS, the observed pattern suggests that higher UPFs consumption may be linked to less balanced diets [5,6,8,41]. Additionally, these findings confirm that UPFs intake declines with age, underscoring the importance of considering these results for future dietary interventions. Additionally, the decline in UPFs intake with age reinforces the importance of targeting dietary interventions across different age groups. Although no direct association with MetS was identified, the findings support the need for public health strategies to reduce UPFs intake.

Nutritional education should play a central role, promoting the consumption of fresh and minimally processed foods while raising the awareness of UPFs-related risks. Regulatory measures to limit UPFs advertising, especially to children and adolescents, and collaboration with the food industry to reformulate products could further support healthier dietary patterns. Ensuring equitable access to nutritious foods is essential for reducing the burden of diet-related metabolic disorders.

This study has several strengths that enhance its validity and relevance. First, the large multicentre cohort, comprising participants from various European countries, constitutes a broad and diverse sample, which significantly increases the generalizability of the results across different socio-cultural and geographical contexts. This diversity makes the study more representative of the overall European population, allowing for the results to be applied to different types of people. Secondly, the methodology used for the dietary data collection, particularly the validated SACANA software, ensured high accuracy when assessing the participants’ food intake. The combination of this methodology with precise measurements of health indicators, such as waist circumference, blood pressure, and lipid and glucose profiles, allowed for a comprehensive assessment of the participants’ nutritional and metabolic status. Moreover, the analyses were conducted with appropriate adjustments for socio-demographic and behavioural factors, adding robustness to the findings and facilitating a more complete understanding of the interaction between diet and metabolic health.

Despite the numerous strengths, the study also has some limitations that must be considered. The potential for selection bias arises from the exclusion of a large portion of the initial sample. Although some significant differences were observed between the included and excluded participants, the differences in UPFs consumption were modest. However, this selection process may have affected the representativeness of our sample and could limit the generalizability of our findings. Having a cross-sectional design, it was not possible to establish causal relationships, limiting the understanding of the long-term effects of UPFs consumption on metabolic syndrome. Moreover, although the study considered socio-demographic and behavioural variables, there remains the issue of factors that were not fully taken into account. Factors, such as physical activity, genetic predisposition, and environmental influences, which could affect the relationship between UPFs consumption and metabolic syndrome, were not considered due to the reduction in the sample size. Another point is that we used a uniform threshold (<500 kcal/day or >3500 kcal/day) to identify implausible energy intake values, applied to all participants regardless of age or sex. This approach, adopted due to the lack of complete data on the participants’ basal metabolic rates and physical activity levels, prevented the use of individualized methods, such as the Goldberg cut-off [42]. While it ensured methodological consistency with our previous study [5], it may have introduced some misclassifications of implausible intakes, particularly among children, adolescents, and men. Finally, interpretations of the presented data should be made with caution, given the methodological limitations of the NOVA classification, including potential subjectivity and misclassification when categorizing certain foods [43]. This limitation suggests that future studies, with differentiated designs, may be needed to identify more subtle effects in younger age groups.

## 5. Conclusions

In conclusion, this study found no association between UPFs consumption and MetS or its individual components in a European cohort. However, a decline in diet quality was observed with increasing UPFs intake, as reflected by the reduced HDAS scores. These findings align with those of previous research, highlighting the complexity of the diet–health relationship. The study emphasizes the need for public health strategies to reduce UPFs consumption and improve diet quality. Further research is recommended to explore the potential implications of UPF-rich diets on metabolic health.

## Figures and Tables

**Table 1:** General characteristics of the study population by age group.

	Age Groups		
Characteristics	Children (6–9 Years) N = 147	Adolescents (10–19 Years) N = 645	Adults ≥ 20 N = 1493
**Age (years**)	9.24 ± 0.50	12.39 ± 1.28	43.54 ± 5.57
**Sex (%)**			
*Male*	48.3	47.8	29.9
*Female*	51.7	52.2	70.1
**BMI (kg m^−2^)**	20.41 ± 3.52	23.09 ± 4.09	26.04 ± 4.78
**WC (cm)**	68.31 ± 9.05	76.43 ± 10.09	86.53 ± 13.07
**SBP (mmHg)**	107.09 ± 7.63	111.90 ± 8.77	117.84 ± 13.63
**DBP (mmHg)**	65.64 ± 5.56	67.30 ± 6.20	75.84 ± 8.86
**TRG (mmol^−1^)**	71.29 ± 31.46	81.88 ± 40.54	93.12 ± 66.47
**HDL–C (mmol^−1^)**	55.28 ± 11.60	51.73 ± 11.54	58.84 ± 15.51
**GLU (mmol^−1^)**	93.33 ± 5.53	95.28 ± 6.33	97.70 ± 16.38
**Income (%)**			
*low*	29.9	33.9	15.2
*low–medium*	10.2	15.1	8.3
*medium*	39.4	34.3	40.1
*medium–high*	6.3	7.0	16.3
*high*	14.2	9.8	20.1
**ISCED (%)**			
*low*	11.1	11.9	3.6
*medium*	52.8	53.0	40.9
*high*	36.1	35.0	55.6
**Country (%)**			
*ITA*	40.8	47.7	11.6
*EST*	2.0	0.9	2.1
*CYP*	7.5	10.1	16.9
*BEL*	14.3	4.5	7.4
*SWE*	12.2	7.0	20.2
*GER*	11.6	17.3	31.7
*HUNG*	8.8	6.2	2.0
*ESP*	2.7	6.2	8.2
**BMI categories (%)**			
*Normal weight*	42.2	39.9	46.9
*Overweight*	35.4	41.1	36.0
*Obese*	22.4	19.1	17.1
**UPFs (%TEI)**			
	48.63 ± 9.64	47.49 ± 10.08	40.53 ± 9.78

Values are expressed as mean ± SD or as number (percentage). BMI, body mass index; WC, waist circumference; SBP, systolic blood pressure; DBP, diastolic blood pressure; TRG, triglyceride; HDL–C, high-density lipoprotein cholesterol; GLU, blood glucose; ISCED, International Standard Classification of Education. Countries: BEL, Belgium; CYP, Cyprus; ESP, Spain; EST, Estonia; GER, Germany; HUNG, Hungary; ITA, Italy; SWE, Sweden. UPFs (%TEI), percentage contribution of ultra-processed foods to total energy intake.

**Table 2:** Associations between quintiles of UPFs intake and MetS and *z*-score for its risk factors for the I.Family cohort (total N = 2285; year 2013/2014).

A	UPFs (%TEI) Quintiles					
Children (6–9 Years)	Q1	Q2	Q3	Q4	Q5	*p* Value
**MetS score**						
Crude	3.48 (2.79–4.2)	3.76 (3.02–4.49)	3.51 (2.82–4.19)	3.70 (2.95–4.45)	3.78 (3.10–4.45)	0.956
Adjusted	3.35 (2.60–4.10)	3.75 (2.99–4.51)	3.69 (3.00–4.39)	3.61 (2.84–4.38)	3.70 (2.92–4.48)	0.938
**WC *z*-score**						
Crude	1.66 (1.23–2.09)	1.73 (1.27–2.19)	1.59 (1.16–2.02)	1.82 (1.42–2.36)	1.90 (1.47–2.32)	0.823
Adjusted	1.59 (1.28–1.89)	1.72 (1.41–2.04)	1.72 (1.43–2.00)	2.01 (1.69–2.33)	1.70 (1.38–2.01)	0.377
**SBP *z*-score**						
Crude	0.38 (0.09–0.69)	0.44 (0.12–0.76)	0.42 (0.12–0.72)	0.25 (0.13–0.52)	0.38 (0.09–0.67)	0.839
Adjusted	0.32 (0.08–0.72)	0.46 (0.16–0.86)	0.50 (0.11–0.87)	0.17 (−0.08–0.42)	0.49 (0.07–0.91)	0.368
**DBP *z*-score**						
Crude	0.47 (0.16–0.76)	0.28 (0.08–0.60)	0.36 (0.06–0.66)	0.15 (−0.25–0.41)	0.30 (0.04–0.59)	0.536
Adjusted	0.50 (0.15–0.85)	0.35 (0.03–0.70)	0.30 (0.02–0.62)	0.17 (−0.18–−0.29)	0.43 (0.07–0.79)	0.204
**TRG *z*-score**						
Crude	0.24 (0.08–0.57)	0.38 (0.22–0.74)	0.35 (0.15–0.68)	0.58 (0.22–0.95)	0.40 (0.13–0.70)	0.738
Adjusted	0.15 (−0.37–−0.67)	0.31 (0.18–0.84)	0.42 (0.18–0.88)	0.58 (0.04–0.97)	0.43 (0.03–0.97)	0.821
**HDL–C *z*-score**						
Crude	−0.38 (−0.71–−0.06)	−0.59 (−0.94–−0.25)	−0.37 (−0.69–−0.04)	−0.57 (−0.92–−0.21)	−0.71 (−0.99–−0.38)	0.533
Adjusted	−0.37 (−0.73–−0.04)	−0.46 (−0.73–−0.08)	−0.44 (−0.78–−0.09)	−0.52 (−0.90–−0.14)	−0.83 (−1.21–−0.44)	0.510
**HOMA index *z*-score**						
Crude	1.08 (0.76–1.39)	1.18 (0.84–1.52)	1.16 (0.85–1.48)	1.09 (0.75–1.44)	1.00 (0.69–1.31)	0.941
Adjusted	1.09 (0.60–1.59)	1.24 (0.73–1.74)	1.15 (0.69–1.61)	1.08 (0.56–1.59)	0.92 (0.40–1.43)	0.930
**B**	**UPFs (%TEI) Quintiles**					
**Adolescents** **(10–19 Years)**	**Q1**	**Q2**	**Q3**	**Q4**	**Q5**	***p* Value**
**MetS score**						
Crude	3.89 (3.57–4.23)	3.96 (3.62–4.29)	3.29 (2.93–3.64)	3.86 (3.48–4.24)	3.32 (2.94–3.69)	0.009
Adjusted	3.84 (3.51–4.16)	3.91 (3.57–4.25)	3.38 (3.04–3.72)	3.94 (3.57–4.31)	3.76 (3.40–4.12)	0.147
**WC *z*-score**						
Crude	1.90 ^a^ (1.72–2.09)	1.94 ^b^ (1.76–2.13)	1.61 (1.42–1.81)	1.83 (1.61–2.04)	1.35 (1.16–1.53)	<0.001
Adjusted	1.64 ^c^ (1.51–1.77)	1.95 (1.81–2.08)	1.73 (1.59–1.86)	1.90 (1.75–2.04)	1.77 (1.62–1.91)	0.011
**SBP *z*-score**						
Crude	0.51 (0.36–0.65)	0.56 (0.42–0.71)	0.48 (0.32–0.63)	0.47 (0.30–0.64)	0.50 (0.33–0.66)	0.920
Adjusted	0.54 (0.35–0.74)	0.55 (0.35–0.75)	0.45 (0.25–0.65)	0.48 (0.26–0.69)	0.51 (0.29–0.72)	0.952
**DBP *z*-score**						
Crude	0.37 (0.22–0.52)	0.49 (0.34–0.64)	0.38 (0.23–0.54)	0.49 (0.32–0.66)	0.51 (0.34–0.69)	0.595
Adjusted	0.48 (0.28–0.68)	0.51 (0.30–0.72)	0.29 (0.08–0.50)	0.52 (0.29–0.75)	0.59 (0.37–0.82)	0.364
**TRG *z*-score**						
Crude	0.47 (0.32–0.62)	0.46 (0.31–0.62)	0.33 (0.17–0.50)	0.50 (0.33–0.69)	0.63 (0.45–0.80)	0.222
Adjusted	0.57 (0.34–0.80)	0.58 (0.35–0.82)	0.41 (0.17–0.65)	0.47 (0.21–0.73)	0.58 (0.33–0.83)	0.795
**HDL–C *z*-score**						
Crude	−0.73 (−0.87–−0.58)	−0.64 (−0.79–−0.50)	−0.45 (−0.61–−0.29)	−0.50 (−0.68–−0.33)	−0.52 (−068–−0.35)	0.079
Adjusted	−0.73 (−0.93–−0.54)	−0.67 (−0.87–−0.46)	−0.50 (−0.71–−0.30)	−0.63 (−0.85–−0.41)	−0.57 (−0.79–−0.36)	0.579
**HOMA index *z*-score**						
Crude	0.96 (0.80–1.11)	0.93 (0.78–1.08)	0.85 (0.69–1.01)	1.05 (0.87–1.22)	0.89 (0.72–1.06)	0.567
Adjusted	1.02 (0.82–1.24)	0.81 (0.60–1.02)	0.82 (0.61–1.04)	1.00 (0.76–1.23)	0.86 (0.64–1.01)	0.479
**C**	**UPFs (%TEI) Quintiles**					
**Adults** **(≥20 Years)**	**Q1**	**Q2**	**Q3**	**Q4**	**Q5**	***p*** **Value**
**WC**						
Crude	88.82 ^b,d^ (87.33–90.30)	85.07 (83.59–86.55)	86.60 (85.06–88.15)	85.30 (83.82–86.79)	86.85 (85.44–88.25)	0.004
Adjusted	87.44 (86.52–88.35)	86.71 (85.82–87.60)	87.29 (86.39–88.21)	86.80 (85.92–87.68)	86.73 (85.81–87.65)	0.704
**SBP**						
Crude	118.39 (116.84–119.95)	116.32 (114.77–117.87)	118.36 (116.74–119.98)	117.74 (116.19–119.30)	118.36 (116.89–119.82)	0.281
Adjusted	117.95 (115.94–119.95)	117.13 (115.17–119.08)	118.80 (116.80–120.80)	118.60 (116.66–120.53)	119.07 (117.06–121.09)	0.683
**DBP**						
Crude	75.99 (74.98–77.00)	74.61 (73.60–75.61)	76.69 (75.64–77.75)	75.72 (74.71–76.73)	76.22 (75.27–77.17)	0.059
Adjusted	76.44 (75.06–77.81)	75.45 (74.11–76.80)	76.25 (74.88–77.62)	75.76 (74.44–77.09)	76.43 (75.05–77.82)	0.798
**TRG**						
Crude	96.82 (89.25–104.40)	90.58 (83.03–98.13)	91.16 (83.26–99.07)	87.67 (80.08–95.25)	98.52 (91.37–105.67)	0.217
Adjusted	92.80 (83.57–102.04)	94.45 (85.43–103.47)	96.89 (87.68–106.09)	94.40 (85.50–103.31)	116.31 (97.02–115.61)	0.275
**HDL–C**						
Crude	56.37 ^b,c,d^ (54.61–58.13)	60.62 (58.87–62.38)	59.85 (58.01–61.69)	59.45 (57.68–61.21)	58.07 (56.41–59.74)	0.008
Adjusted	57.19 (54.96–59.42)	59.16 (56.99–61.34)	58.20 (55.98–60.42)	56.45 (54.30–58.60)	55.81 (53.57–58.05)	0.232
**GLU**						
Crude	98.94 (97.07–101.80)	93.38 (96.52–100.23)	96.45 (94.51–98.40)	95.97 (94.10–97.83)	98.57 (96.80–100.32)	0.091
Adjusted	99.64 (97.28–101.99)	98.81 (96.51–101.11)	98.50 (96.15–100.84)	96.13 (93.86–98.40)	101.07 ^e^ (98.70–103.45)	0.047

Values are expressed as mean ± SD. WC, waist circumference; SBP, systolic blood pressure; DBP, diastolic blood pressure; TRG, triglyceride; HDL–C, high-density lipoprotein cholesterol; HOMA, homeostatic model assessment; MetS, metabolic syndrome; GLU, blood glucose. Analysis adjusted for sex, age, country, family income, family ISCED, BMI, and total daily energy intake. Multiple comparison (Bonferroni post hoc test). Values are expressed as mean (95%CI). ^a^ *p* <0.005, Q1 vs. Q5; ^b^ *p* < 0.001, Q2 vs. Q5; ^c^ *p* < 0.05, Q1 vs. Q2; ^d^ *p* < 0.005, Q1 vs. Q4; ^e^ *p* < 0.005, Q5 vs. Q4.

**Table 3:** Odds ratios of MetS or MetS risk factors across UPFs quintiles in children (**A**), adolescents (**B**), and adults (**C**) from the I.Family cohort.

A				
Children (6–9 Years)	Q2 OR (95%CI)	Q3 OR (95%CI)	Q4 OR (95%CI)	Q5 OR (95%CI)
MetS score > 90 percentile	0.94 (0.13–6.80)	0.83 (0.12–5.93)	0.52 (0.07–4.01)	0.51 (0.06–4.13)
WC *z*-score > 90 percentile	1.24 (0.21–7.17)	0.46 (0.08–2.73)	0.53 (0.09–3.27)	0.14 (0.02–1.01)
SBP *z*-score > 90 percentile	0.88 (0.10–7.78)	0.63 (0.06–6.33)	0.12 (0.02–2.30)	0.65 (0.07–6.30)
DBP *z*-score > 90 percentile	0.25 (0.06–1.03)	0.37 (0.14–1.54)	0.28 (0.06–1.35)	0.21 (0.04–1.10)
TRG *z*-score > 90 percentile	0.53 (0.11–2.52)	0.77 (0.14–4.18)	0.66 (0.12–3.56)	0.19 (0.03–1.29)
HDL–C *z*-score < 10 percentile	2.52 (0.35–8.89)	2.35 (0.66–8.95)	2.55 (0.77–10.03)	2.65 (1.76–11.14)
HOMA index *z*-score > 90 percentile	1.76 (0.33–9.37)	0.44 (0.08–2.39)	0.32 (0.05–1.95)	0.53 (0.04–1.54)
**B**				
**Adolescents** **(10–19 Years)**	**Q2** **OR (95%CI)**	**Q3** **OR (95%CI)**	**Q4** **OR (95%CI)**	**Q5** **OR (95%CI)**
MetS score > 90 percentile	0.75 (0.31–1.82)	0.42 (0.15–1.19)	1.14 (0.45–2.89)	1.14 (0.45–2.89)
WC *z*-score > 90 percentile	0.49 (0.24–1.01)	0.39 (0.17–1.89)	0.52 (0.22–1.24)	0.45 (0.18–1.11)
SBP *z*-score > 90 percentile	1.51 (0.64–3.56)	0.57 (0.20–1.66)	1.16 (0.42–3.16)	1.10 (0.40–3.05)
DBP *z*-score > 90 percentile	1.80 (0.81–3.98)	1.31 (0.56–3.08)	1.06 (0.40–2.77)	1.09 (0.41–2.86)
TRG *z*-score > 90 percentile	0.95 (0.38–2.37)	0.88 (0.35–2.21)	1.29 (0.50–3.32)	1.16 (0.44–3.07)
HDL–C *z*-score < 10 percentile	0.90 (0.41–1.96)	0.37 (0.14–1.01)	0.71 (0.28–1.78)	0.68 (0.25–1.85)
HOMA index *z*-score > 90 percentile	1.42 (0.72–2.79)	1.15 (0.56–2.35)	1.15 (0.53–2.52)	1.04 (0.45–2.39)
**C**				
**Adults** **(≥20 Years)**	**Q2** **OR (95%CI)**	**Q3** **OR (95%CI)**	**Q4** **OR (95%CI)**	**Q5** **OR (95%CI)**
MetS	0.96 (0.41–2.28)	0.56 (0.21–1.49)	0.28 (0.08–0.94)	0.80 (0.32–2.00)
Elevated WC	1.04 (0.60–1.82)	1.14 (0.65–2.00)	1.17 (0.67–2.07)	1.08 (0.60–1.93)
Elevated BP	0.61 (0.35–1.06)	0.59 (0.33–1.03)	0.84 (0.49–1.43)	0.83 (0.49–1.42)
Elevated GLU	1.23 (0.66–2.30)	1.05 (0.54–2.03)	1.00 (0.50–1.93)	1.25 (0.65–1.99)
Elevated TRG	0.72 (0.39–1.31)	0.63 (0.34–1.17)	0.92 (0.53–1.71)	0.77 (0.42–1.41)
Low HDL–C	0.54 (0.34–0.85)	0.78 (0.50–1.22)	0.82 (0.53–1.28)	0.91 (0.58–1.42)

Values are expressed as odds ratio and 95% confidence intervals. MetS, metabolic syndrome; WC, waist circumference; SBP, systolic blood pressure; DBP, diastolic blood pressure; TRG, triglyceride; HDL–C, high-density lipoprotein cholesterol; HOMA, homeostatic model assessment; BP, blood pressure; GLU, blood glucose. Analysis adjusted for sex, age, country, family income, family ISCED, BMI, and total daily energy intake.

## Data Availability

The original contributions presented in the study are included in the article/Supplementary Material, further inquiries can be directed to the corresponding author.

## Supplementary Materials

The following supporting information can be downloaded at: https://www.mdpi.com/article/10.3390/nu17132252/s1, Table S1. General characteristics of the study population by quintiles of UPF intake (%TEI) (Total N = 2285; year 2013/2014).
