# Supplementary material for: Ultra-Processed Foods Consumption and Metabolic Syndrome in European Children, Adolescents, and Adults: Results from the I.Family Study
Source: Nutrients. 2025 Jul 7;17(13):2252. doi: 10.3390/nu17132252 (PMC12251691; doi:10.3390/nu17132252)
Supplement: Supplementary file 1 [file nutrients-17-02252-s001.zip › nutrients-3679992-supplementary.pdf]

Table S1. General characteristics of the study population by quintiles of UPF intake (%TEI) (Total N = 2285; year 2013/2014).

A	UPFs (%TEI) Quintiles				
**Characteristics**	**Q1 (*n* = 31)**	**Q2 (*n* = 27)**	**Q3 (*n* = 31)**	**Q4 (*n* = 26)**	**Q5 (*n* = 32)**
**Children (6–9 Years)**					
**Age (ys)**	9.32 ± 0.47	9.23 ± 0.52	9.19 ± 0.48	9.14 ± 0.49	9.30 ± 0.54
**Sex(%)**					
Male	51.6	40.7	38.7	53.8	56.3
Female	48.4	59.3	61.3	46.2	43.8
**BMI z-score**	3.32 ± 2.19	3.02 ± 2.34	2.72 ± 2.23	3.20 ± 2.34	3.30 ± 2.53
**WC z-score**	1.66 ± 1.17	1.73 ± 1.36	1.59 ± 1.16	1.89 ± 1.27	1.90 ± 1.11
**SBP z-score**	0.39 ± 0.73	0.44 ± 0.94	0.42 ± 0.69	0.19 ± 0.98	0.38 ± 0.85
**DBP z-score**	0.46 ± 0.96	0.28 ± 0.71	0.36 ± 0.70	0.08 ± 0.92	0.30 ± 0.89
**TRG z-score**	0.24 ± 0.90	0.38 ± 0.97	0.35 ± 0.97	0.58 ± 0.92	0.36 ± 0.94
**HDL–C z-score**	-0.38 ± 0.77	-0.59 ± 0.97	-0.37 ± 0.98	-0.57 ± 0.90	-0.71 ± 0.93
**HOMA index z-score**	1.07 ± 1.03	1.18 ± 0.99	1.16 ± 0.76	1.09 ± 0.84	1.00 ± 0.81
**Income (%)**					
low	40.0	44.0	11.1	27.3	28.6
low–medium	8.0	28.0	7.4	4.5	3.6
medium	44.0	4.0	40.7	50.0	57.1
medium–high	8.0	4.0	7.4	9.1	3.6
high	0.0	20.0	33.3	9.1	7.1
**ISCED (%)**					
low	0.0	15.4	12.9	16.0	12.9
medium	71.0	57.7	35.5	44.0	54.8
high	29.0	26.9	51.6	40.0	32.3
**Country (%)**					
ITA	58.1	55.6	29	34.6	28.1
EST	0	3.7	3.2	0	3.1
CYP	9.7	7.4	3.2	7.7	9.4
BEL	9.7	7.4	22.6	11.5	18.8
SWE	6.5	11.1	19.4	19.2	6.3
GER	3.2	11.1	12.9	15.4	15.6
HUNG	6.5	3.7	6.5	7.7	18.8
ESP	6.5	0.0	3.2	3.8	0.0
**BMI categories (%)**					
Normal weight	38.7	44.4	54.8	38.5	34.4
Overweight	45.2	22.2	25.8	38.5	43.8
Obese	16.1	33.3	19.4	23.1	21.9
**MetS score**	3.48 ± 2.08	3.76 ± 1.95	3.51 ± 1.90	3.70 ± 1.97	3.78 ± 1.75
**MetS score > 90th percentile (*n* = 27)**	18.5	18.5	18.5	18.5	25.9
**HDAS**	18.90 ± 6.80	17.79 ± 6.76	17.67 ± 7.52	17.27 ± 4.56	15.79 ± 5.09
**B**	**UPFs (%TEI) Quintiles**				
**Characteristics**	**Q1 (*n* = 149)**	**Q2 (*n* = 146)**	**Q3 (*n* = 127)**	**Q4 (*n* = 109)**	**Q5 (*n* = 114)**
**Adolescents (10–19 Years)**					
**Age (ys)**	12.28 ± 1.34	12.51 ± 1.22	12.42 ± 1.30	12.31 ± 1.21	12.44 ± 1.35
**Sex (%)**					
Male	48.3	47.3	45.7	51.4	46.5
Female	51.7	52.7	54.3	48.6	53.5
**BMI z-score**	3.48 ± 2.03	3.52 ± 2.13	3.13 ± 2.43	3.36 ± 2.28	2.36 ± 2.49
**WC z-score**	1.90 ± 1.14	1.94 ± 1.04	1.61 ± 1.17	1.83 ± 1.12	1.35 ± 1.22

**SBP z-score**	0.51 ± 0.91	0.56 ± 0.96	0.48 ± 0.80	0.47 ± 0.91	0.49 ± 0.85
**DBP z-score**	0.37 ± 0.91	0.49 ± 0.95	0.38 ± 0.88	0.49 ± 0.96	0.51 ± 0.84
**TRG z-score**	0.47 ± 0.99	0.46 ± 0.97	0.33 ± 0.90	0.51 ± 0.94	0.63 ± 0.98
**HDL–C z-score**	-0.73 ± 0.93	-0.64 ± 0.87	-0.45 ± 0.86	-0.50 ± 1.04	-0.51 ± 0.90
**HOMA index z-score**	0.95 ± 0.93	0.93 ± 0.92	0.85 ± 1.00	1.05 ± 0.91	0.89 ± 0.93
**Income (%)**					
low	37.3	35.2	37.6	28.9	27.1
low–medium	15.1	21.1	11.0	14.5	11.8
medium	31.0	29.7	34.9	42.2	37.6
medium–high	4.8	7.0	7.3	4.8	11.8
high	11.9	7.0	9.2	9.6	11.8
**ISCED (%)**					
low	11.0	11.3	12.0	14.0	11.9
medium	55.5	57.4	52.0	51.4	46.8
high	33.6	31.2	36.0	34.6	41.3
**Country (%)**					
ITA	56.4	57.6	57.5	38.5	21.9
EST	0.7	0.7	0.8	0.9	1.8
CYP	12.8	12.5	6.3	9.2	8.8
BEL	2.7	2.8	4.7	5.5	7.9
SWE	5.4	4.2	6.3	11.0	9.6
GER	8.1	11.8	11.0	22.9	37.7
HUNG	7.4	4.9	4.7	6.4	7.9
ESP	6.7	5.6	8.7	5.5	4.4
**BMI categories (%)**					
Normal weight	36.2	34.0	40.2	34.9	56.1
Overweight	36.2	48.6	44.1	46.8	28.9
Obese	27.5	17.4	15.7	18.3	14.9
**MetS score**	3.90 ± 2.22	3.95 ± 2.00	3.29 ± 1.88	3.86 ± 1.97	3.31 ± 2.07
**MetS score > 90th percentile (*n* = 63)**	28.6	22.2	12.7	20.6	15.9
**HDAS**	20.03 ± 7.29	19.11 ± 7.53	18.94 ± 6.88	18.39 ± 7.31	16.63 ± 8.04
**C**	**UPFs (%TEI) Quintiles**				
**Characteristics**	**Q1 (*n* = 296)**	**Q2 (*n* = 298)**	**Q3 (*n* = 272)**	**Q4 (*n* = 295)**	**Q5 (*n* = 332)**
**Adults ≥ 20 years**					
**Age (ys)**	44.67 ± 5.84	43.62 ± 5.64	43.46 ± 5.48	43.41 ± 5.38	42.64 ± 5.33
**Sex (%)**					
Male	29.7	28.5	28.7	31.2	31.3
Female	70.3	71.5	71.3	68.8	68.7
**BMI (kg m^{-2})**	26.98 ± 5.43	25.44 ± 4.31	26.17 ± 4.66	25.46 ± 4.33	26.16 ± 4.88
**WC (cm)**	88.81 ± 14.16	85.07 ± 12.42	86.60 ± 12.53	85.30 ± 12.38	86.85 ± 13.42
**SBP (mmHg)**	118.39 ± 14.32	116.32 ± 12.58	118.36 ± 12.57	117.75 ± 14.54	118.36 ± 13.88
**DBP (mmHg)**	76.00 ± 9.34	74.61 ± 8.50	76.69 ± 8.16	75.72 ± 9.28	76.22 ± 8.83
**TRG (mmol^{-1})**	95.82 ± 60.52	90.58 ± 61.17	91.17 ± 64.27	87.67 ± 47.17	99.52 ± 88.67
**HDL–C (mmol^{-1})**	56.37 ± 15.62	60.63 ± 15.88	59.85 ± 15.41	59.45 ± 15.55	58.08 ± 14.88
**GLU (mmol^{-1})**	98.94 ± 17.15	98.38 ± 17.73	96.45 ± 12.27	95.97 ± 11.21	99.55 ± 20.68
**Income (%)**					
low	17.8	20.2	15.0	11.4	12.1
low–medium	8.9	9.0	6.9	8.5	8.1
medium	37.1	37.5	40.7	42.8	42.4
medium–high	12.7	13.5	19.1	19.2	16.8
high	23.6	19.9	18.3	18.1	20.5
**ISCED (%)**					

low	5.3	3.5	2.3	2.7	4.0
medium	39.9	41.3	44.5	39.9	39.3
high	54.8	55.2	53.2	57.4	56.7
**Country (%)**					
ITA	11.5	15.8	13.2	13.6	4.8
EST	1.0	3.4	0.7	1.7	3.3
CYP	30.7	20.5	11.4	12.2	9.9
BEL	3.7	4.4	7.0	7.1	13.9
SWE	14.9	21.1	21.7	21.7	21.4
GER	22.6	22.1	37.9	34.2	41.3
HUNG	2.4	2.0	1.5	2.7	1.5
ESP	13.2	10.7	6.6	6.8	3.9
**BMI categories (%)**					
Normal weight	43.2	50.0	43.8	50.2	47.0
Overweight	34.1	36.2	37.5	36.6	35.5
Obese	22.6	13.8	18.8	13.2	17.5
**MetS (*n* = 55)**					
Yes	32.7	20.0	14.5	7.3	25.5
**HDAS**					
	28.08 ± 8.96	27.15 ± 8.53	26.38 ± 8.76	25.59 ± 9.07	24.17 ± 9.12

Values are expressed as mean ± SD or as number (percentage). %TEI, percentage contribution to total energy intake; BMI, body mass index; WC,waist circumference; SBP, systolic blood pressure; DBP, diastolic blood pressure; TRG, triglyceride; HDL–C, high–density lipoprotein cholesterol; HOMA, homeostatic model assessment; GLU, blood glucose; ISCED, International Standard Classification of Education; Countries: BEL, Belgium; CYP, Cyprus; ESP, Spain; EST, Estonia; GER, Germany; HUNG, Hungary; ITA, Italy; SWE, Sweden; MetS score, metabolic syndrome score; HDAS, Healthy Dietary Adherence Score.
